# DNA Repair Expression Profiling to Identify High-Risk Cytogenetically Normal Acute Myeloid Leukemia and Define New Therapeutic Targets

**DOI:** 10.3390/cancers12102874

**Published:** 2020-10-06

**Authors:** Ludovic Gabellier, Caroline Bret, Guillaume Bossis, Guillaume Cartron, Jérôme Moreaux

**Affiliations:** 1 Département d’Hématologie Clinique, CHU Montpellier, University of Montpellier, 34395 Montpellier, France; ludovic.gabellier@igmm.cnrs.fr (L.G.); g-cartron@chu-montpellier.fr (G.C.); 2 UFR de Médecine, University of Montpellier, 34003 Montpellier, France; c-bret@chu-montpellier.fr; 3 Institut de Génétique Moléculaire de Montpellier (IGMM), University of Montpellier, CNRS, 34090 Montpellier, France; guillaume.bossis@igmm.cnrs.fr; 4 CHU Montpellier, Department of Biological Hematology, 34395 Montpellier, France; 5 Institute of Human Genetics, IGH, CNRS, University of Montpellier, 34395 Montpellier, France; 6 Equipe Labellisée Ligue Contre le Cancer, 75013 Paris, France; 7 Institut Universitaire de France (IUF), 75005 Paris, France

**Keywords:** acute myeloid leukemia, normal karyotype, DNA repair, risk score, precision medicine

## Abstract

**Simple Summary:**

Acute myeloid leukemia (AML) is the second most frequent type of adult leukemias. Cytogenetically normal acute myeloid leukemias represent about 50% of total adult AML, exhibit no chromosomal abnormalities, and present high heterogeneity regarding the clinical outcome. Deregulation of DNA repair mechanisms is involved in the adaptation of cancer cells to replicative stress and resistance to genotoxic agents. We investigate the prognostic value of genes related to the major DNA repair pathways. The data reveals specific patterns of gene expression in CN-AML that have prognostic value. Combined with *NPM1* and *FLT3* mutational status, our gene expression-based DNA repair score might be used as a biomarker to predict outcomes for patients with CN-AML. DNA repair score has the potential to identify CN-AML patients whose tumor cells are dependent on specific DNA repair pathways to design new targeted therapies.

**Abstract:**

Cytogenetically normal acute myeloid leukemias (CN-AML) represent about 50% of total adult AML. Despite the well-known prognosis role of gene mutations such as *NPM1* mutations of *FLT3* internal tandem duplication (*FLT3*-ITD), clinical outcomes remain heterogeneous in this subset of AML. Given the role of genomic instability in leukemogenesis, expression analysis of DNA repair genes might be relevant to sharpen prognosis evaluation in CN-AML. A publicly available gene expression profile dataset from two independent cohorts of patients with CN-AML were analyzed (GSE12417). We investigated the prognostic value of 175 genes involved in DNA repair. Among these genes, 23 were associated with a prognostic value. The prognostic information provided by these genes was summed in a DNA repair score, allowing to define a group of patients (*n* = 87; 53.7%) with poor median overall survival (OS) of 233 days (95% CI: 184–260). These results were confirmed in two validation cohorts. In multivariate Cox analysis, the DNA repair score, *NPM1,* and *FLT3*-ITD mutational status remained independent prognosis factors in CN-AML. Combining these parameters allowed the identification of three risk groups with different clinical outcomes in both training and validation cohorts. Combined with *NPM1* and *FLT3* mutational status, our GE-based DNA repair score might be used as a biomarker to predict outcomes for patients with CN-AML. DNA repair score has the potential to identify CN-AML patients whose tumor cells are dependent on specific DNA repair pathways to design new therapeutic avenues.

## 1. Introduction

Acute myeloid leukemia (AML) is the second most frequent type of adult leukemias. When analyzed with conventional cytogenetics, about 40–50% of AML exhibits no chromosomal abnormalities and are defined as “cytogenetically normal AML” (CN-AML) [1]. Recurrently mutated genes in CN-AML were identified, such as *NPM1*, signal transduction genes (*FLT3*), or myeloid transcription factor genes (*CEBPA, RUNX1*) [2]. Based on the presence, absence, and allelic ratio of these mutations, CN-AML may be classified in favorable, intermediate, or adverse prognosis, illustrating the high heterogeneity of clinical outcomes in this AML subset [3]. Yet, a wide diversity of gene mutations occurring in CN-AML were revealed by deep sequencing techniques, such as mutations of DNA modification, cohesin or tumor-suppressor genes, suggesting the wide heterogeneity of molecular mechanisms involved in leukemogenesis [4,5,6].

Even if the study of the mutational landscape by new DNA sequencing technologies demonstrated a low mutation frequency in AML compared to other cancers [7], genomic instability remains a well-described leukemogenesis mechanism, illustrated by the high frequency of AML with non-random cytogenetics abnormalities or with complex karyotype [8,9]. Therefore, the role of DNA damage response (DDR) in the AML field has been widely studied. Polymorphic variants of genes involved in several DNA repair pathways had been associated with the onset of AML, such as XPD-Lys751Gln, involved in the nucleotide excision repair (NER) mechanism [10]. Recurrent AML fusion transcripts such as RUNX1-RUNX1T1 or PML-RARA has also been demonstrated to downregulate the expression of genes implied in DDR [11,12,13,14]. Moreover, children or young adults AML are often associated with hereditary diseases due to DNA repair gene mutations, such as Fanconi disease [15], Bloom syndrome, or Werner syndrome [16]. Finally, dysregulation in DDR also contributes to increased resistance to conventional chemotherapy by several mechanisms, such as paradoxically increased expression of DDR or cell cycle check-point genes [17,18,19].

FLT3 and NPM1 have also been shown to play a role in the DNA damage response in AML. *FLT3*-ITD mutations, occurring in about 20–25% of CN-AML, leads to constitutive activation of FLT3 and therefore confers a growth advantage to leukemic cells. Several studies showed that the level of reactive oxygen species (ROS) was increased in *FLT3*-ITD mutated AML cells and correlated with high levels of double-strand break (DSB) and lower efficiency of non-homologous end joining (NHEJ) repair pathway [20]. Moreover, the use of tyrosine kinase inhibitors may reduce both ROS and DSB levels, and increase DNA repair efficiency, overcoming the chemo-resistance of these cells [20,21]. Other mechanisms have been suggested to explain the role of *FLT3*-ITD in DNA damages and acquired drug resistance of AML cells, such as telomere-related genome instability [22], or paradoxical up-regulation of RAD51 [23]. *NPM1* is the most commonly mutated gene in CN-AML, with more than 50 described mutations. The prognostic significance of these mutations and co-mutations in other genes has been widely studied [24]. The role of NPM1 in DNA damage response and maintenance of genome stability is less clear. Nevertheless, NPM1 is involved in the regulation of centrosome duplication during the cell cycle [25] or is recruited in its phosphorylated form (NPM1-pT199) on DSB foci, even if its role in DSB repair remains discussed [26]. NPM1 is also involved in the regulation of key DNA repair factors, such as APEX1 or TP53 [27,28]. Therefore, *NPM1* mutations in AML result in APEX1 abnormal cytoplasmic accumulation, and impaired base excision repair (BER) activity [29], potentially explaining a chemotherapy improved response in *NPM1*-mutated AML.

In the current study, we investigate the prognostic value of genes related to the major DNA repair pathways. The data reveals specific patterns of gene expression in CN-AML that have prognostic value. Therefore, the expression analysis of DNA repair genes might be relevant in the context of CN-AML to sharpen prognosis evaluation of this heterogeneous AML subset.

## 2. Results

### 2.1. Linking Expression of DNA Repair Genes and AML Patient Overall Survival

Considering the important role of DNA repair in drug resistance and adaptation to replication stress in cancer cells, we first aimed to identify the DNA repair genes associated with overall survival in CN-AML. A list set of 175 genes involved in six major DNA repair pathways—ER, NER, mismatch repair (MMR), homologous recombination repair (HRR), NHEJ, and Fanconi (FANC) pathways—defined using the REPAIRtoire database (http://repairtoire.genesilico.pl) and review of the literature (Table S1). Using the MaxStat R function (https://cran.r-project.org/web/packages/maxstat/index.html), we identified 23 out of the 175 genes whose level of expression had a prognostic value in the two independent cohorts. This approach allowed the selection of DNA repair genes that could provide a selective advantage to CN-AML cells through adaptation to replicative stress and chemoresistance. Among them, a high expression of 19 genes was associated with a poor outcome (Table 1). No statistically significant prognostic value was found for any gene involved in the NHEJ pathway.

**Figure 1:**
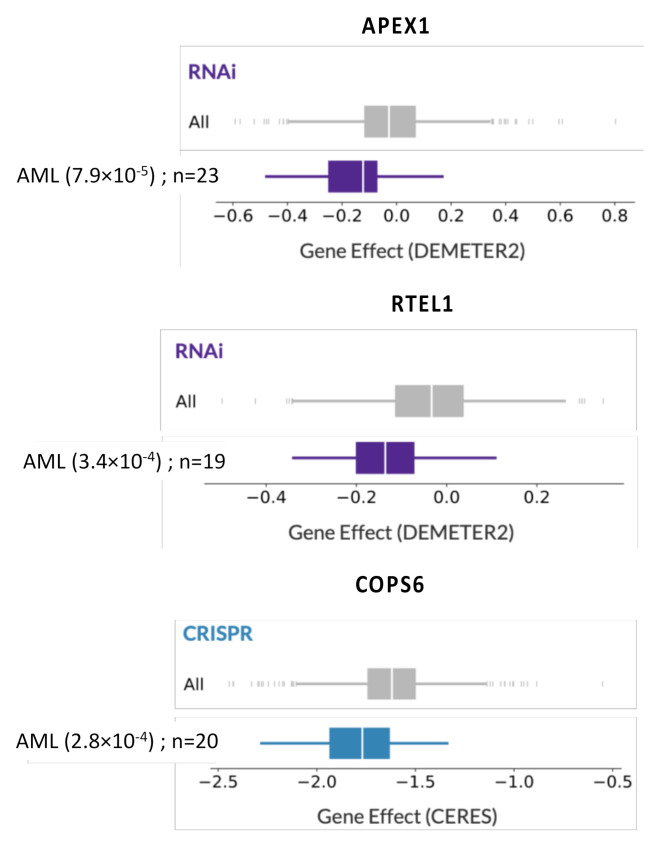
The silencing of *APEX1*, *RTEL1,* and *COPS6* impairs acute myeloid leukemia (AML) cell growth. Using CRISPR or RNAi screening publicly available data (Dependency Map data, Broad Institute, www.depmap.org), dependency scores of *APEX1*, *RTEL1,* and *COPS6* underline their specific importance for AML cell survival compared to all cell lines tested.

To further corroborate gene expression data on a functional level, we studied CRISPR or RNAi screening publicly available data (Dependency Map data, Broad Institute, www.depmap.org) [30,31]. Interestingly, among the 19 genes associated with a poor outcome, *APEX1* (BER), *RTEL1* (HRR), and *COPS6* (NER) were identified as significant essential AML genes (*p* = 7.9 × 10^−5^, 3.4 × 10^−4^, and 2.8 × 10^−4^ respectively) (Figure 1).

### 2.2. GEP-Based DNA Repair Score for Predicting CN-AML Patients’ Survival

Then, we searched to combine the prognostic information of these genes in a GE-based DNA repair risk score. The 23 DNA repair genes associated with a prognostic value included 4 coding genes for the BER pathway, 6 genes for the FANC pathway, 6 genes for the HRR pathway, 3 genes for the MMR pathway, and 8 genes for the NER pathway (Table 1). Four out of these 23 probe sets (BRCA2, ERCC1, PMS2/PMS2CL, and XRCC1) were involved in two different pathways. A specific GE-based risk score was established for BER, FANC, HRR, MMR, and NER DNA repair pathways. GE-based DNA repair scores were defined by the sum of the beta coefficients of the Cox model for each prognostic gene, weighted by +1 or −1 according to the patient signal above or below/equal the probe set MaxStat value as previously described [32,33,34,35]. Using the Maxstat R function, high BER, FANC, HRR, MMR, and NER score values were significantly associated with poor prognosis in the training cohort (Supplementary Figure S1).

In Cox multivariate analysis, only HRR and NER scores remained associated with overall survival in the training cohort (Table 2). Therefore, a global DNA repair score was established, incorporating the prognostic value of HRR and NER scores. To this aim, CN-AML patients were split into three subgroups: group I included patients with low NER and HRR risk score values (*n* = 20), group III included patients with high NER and HRR risk scores (*n* = 87), and group II included patients with NER or HRR high-risk score value (*n* = 55).

**Figure 2:**
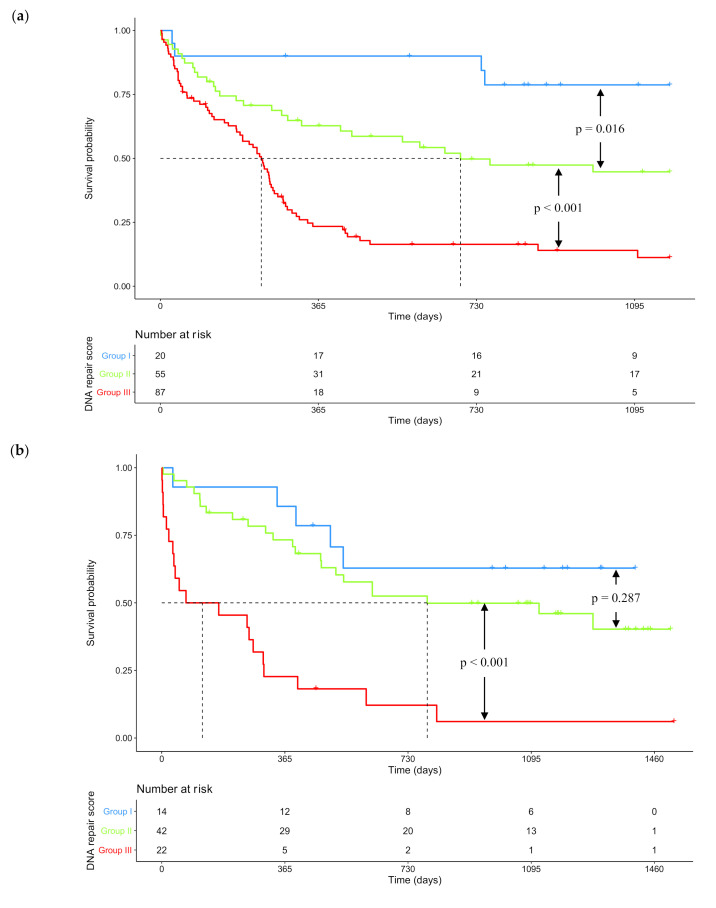
Kaplan–Meier survival curves according to risk stratification determined by DNA repair score. (**a**) Kaplan–Meier survival curve in the training cohort (*n* = 162). Median OS was not reached (95% CI: NR-NR), 693 days (95% CI: 414–NR) and 233 days (95% CI: 184–260) respectively for patients in groups I (low DNA repair score), II (medium DNA repair score) and III (high DNA repair score). One-year OS was 90.0% (95% CI: 77.7–100) in group I, 62.8% (95% CI: 51.1–77.2) in group II, and 23.4% (95% CI: 15.8–34.7) in group III. (**b**) Kaplan–Meier survival curve in the validation cohort (*n* = 78). Median OS was not reached (95% CI: 538-NR), 787 days (95% CI: 473-NR) and 120 days (95% CI: 36–303) respectively for patients in groups I (low DNA repair score), II (medium DNA repair score) and III (high DNA repair score). One-year OS was 85.7% (95% CI: 69.2–100) in group I, 73.3% (95% CI: 60.9–88.2) in group II, and 22.7% (95% CI: 10.5–49.1) in group III. *p*-values were determined with the log-rank test. NR: not reached.

After a median follow-up of 1176 days (95% CI: 916-NR), the median overall survival (OS) was 293 days (95% CI: 252–461) for the whole training cohort (Figure S2a). One-year OS was 45.2% (95% CI: 38.0–53.8). According to risk groups determined by the DNA repair score, median OS was not reached (95% CI: NR-NR), 693 days (95% CI: 414-NR) and 233 days (95% CI: 184–260) respectively for patients in groups I, II, and III (Figure 2a). Median OS were statistically different between each risk group (log-rank test; *p* = 0.016 between group I and II; *p* < 0.001 between group II and III).

We searched to validate these results in an independent cohort of 78 patients. HRR and NER scores computed with training cohort parameters were also prognostic in this validation cohort (Table S2). The global DNA repair score was also computed. In the validation set, risk groups included 14, 42, and 22 patients respectively in groups I, II, and III. After a median follow-up of 1183 days (95% CI: 1092–1383), the median overall survival (OS) was 538 days (95% CI: 388–1278) for the whole validation cohort (Figure S2b). One-year OS was 61.1% (95% CI: 51.1–73.0). According to risk groups determined by the DNA repair score, median OS was not reached (95% CI: 538-NR), 787 days (95% CI: 473-NR) and 120 days (95% CI: 36–303), respectively for patients in groups I, II, and III (Figure 2b). Even if survival analysis failed to demonstrate a statistical difference between groups I and II (log-rank test; *p* = 0.287), OS was still statistically different between risk groups II and III (log-rank test; *p* < 0.001).

We also validated the prognostic value of the DNA repair score in another independent cohort (Verhaak cohort) with CN-AML [36,37,38] using the same parameters defined within the training cohort. Of major interest, the DNA repair score was also a significant prognostic factor in this cohort of 181 patients (*p* = 0.03) for overall survival (Figures S2c and S3a). Furthermore, the DNA repair score was also a poor prognostic factor for event-free survival (EFS) (Figure S3b) suggesting an association of DNA repair score with disease relapse (events were defined as progressive disease, relapse, or death).

There was no significant difference between subgroups according to age in these three cohorts (Figure S4). Altogether, these data underlined the identification of high-risk CN-AML patients characterized by DNA repair dysregulation and that might benefit from DNA repair targeted therapies.

### 2.3. DNA Repair Score and NPM1 / FLT3 Mutational Status Combination as Prognosis Factors in CN-AML

Because *NPM1* mutations and *FLT3*-ITD (internal tandem duplication) are well-described prognosis factors in CN-AML, we conducted another Cox analysis to determine whether our DNA repair score provides additional prognostic information. Prognostic classification according to *NPM1* and *FLT3* mutational status was established in both cohort according to actual recommendations [3]: patients with only *NPM1* mutation were classified as “better outcome”, patients with only *FLT3*-ITD were classified as “adverse prognosis” and patients with both or none of these mutations were classified as “intermediate prognosis”. Kaplan–Meier survival curves according to *NPM1* and *FLT3* mutational status are presented in Supplementary Figure S5 for both training and validation cohorts.

Using multivariate Cox analysis, our DNA repair score and *NPM1*/*FLT3* mutation classification remained independently associated with survival (Table 3 and Table S3). Therefore, we investigated the interest of combining DNA repair score and *NPM1* / *FLT3* mutational status to predict CN-AML outcome. Patients were classified according to prognosis value of DNA repair score (0 point for group I; 1 for group II; 2 for group III), and *NPM1* / *FLT3* mutational status (0 point if *NPM1* mutated without *FLT3*-ITD; 2 points if *FLT3*-ITD without *NPM1* mutation; 1 point in other situations). The sum of the prognostic information was computed for all patients, splitting the training cohort in five groups. When the Kaplan–Meier analysis did not show a significant OS difference between consecutive groups, we merged the two groups. This approach resulted in three prognostic groups with significant different OS values: group A for patients with 0 or 1 point, group B for patients with 2 points, and group C for patients with 3 or 4 points. (Table 4 and Figure S6).

**Figure 3:**
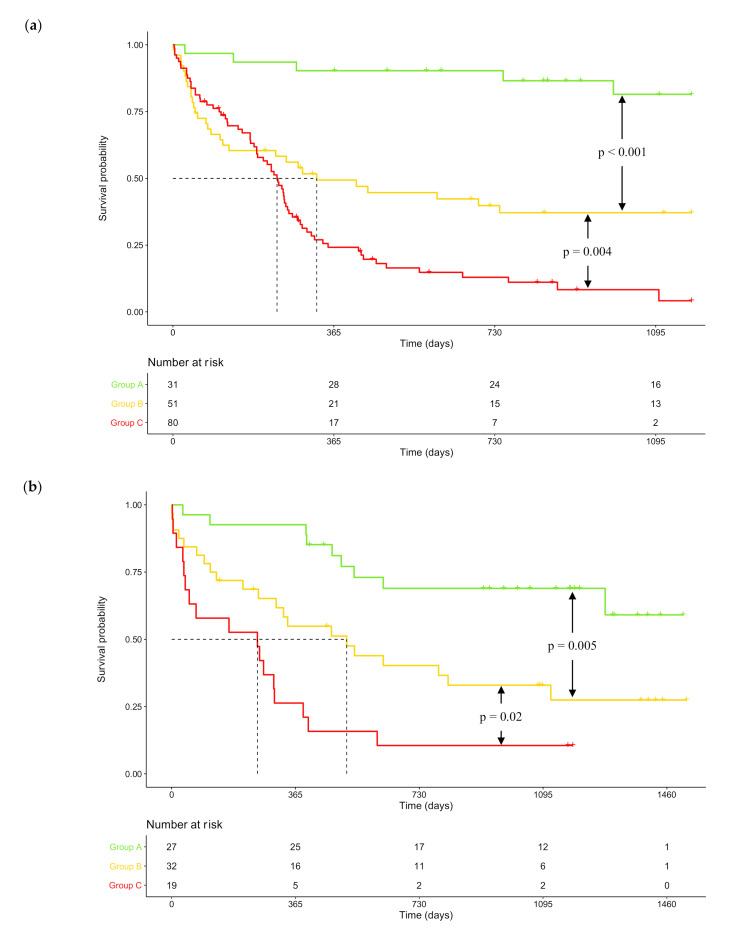
Kaplan–Meier survival curves according to risk groups determined by combined score incorporating DNA repair score and *NPM1*/*FLT3* mutational status. (**a**) Kaplan–Meier survival curve in the training cohort (*n* = 162). Median OS was not reached (95% CI: NR-NR), 326 days (95% CI: 127–NR) and 236 days (95% CI: 190–263) respectively for patients in groups A, B and C. One-year OS was 90.3% (95% CI: 80.5–100) in group A, 49.3% (95% CI: 37.1–65.7) in group B, and 24.2% (95% CI: 16.2–36.2) in group C. (**b**) Kaplan–Meier survival curve in the validation cohort (*n* = 78). Median OS was not reached (95% CI: 1278-NR), 516 days (95% CI: 308-NR) and 253 days (95% CI: 52–403) respectively for patients in groups A, B and C. One-year OS was 92.6% (95% CI: 83.2–100) in group A, 54.9% (95% CI: 39.8–75.7) in group B, and 26.5% (95% CI: 12.4–55.8) in group C. *p*-values were determined with the log-rank test. NR: not reached.

In the training cohort, median OS was not reached (95% CI: NR-NR), 326 days (95% CI: 127-NR) and 236 days (95% CI: 190–263) respectively for patients in groups A, B, and C. One-year OS was 90.3% (95% CI: 80.5–100) in group A, 49.3% (95% CI: 37.1–65.7) in group B and 24.2% (95% CI: 16.2–36.2) in group C. These results were confirmed in the validation cohort where median OS was not reached (95% CI: 1278-NR), 516 days (95% CI: 308-NR) and 253 days (95% CI: 52–403) for patients respectively in groups A, B, and C. One-year OS was 92.6% (95% CI: 83.2–100) in group A, 54.9% (95% CI: 39.8–75.7) in group B and 26.5% (95% CI: 12.4–55.8) in group C. OS was statistically different between groups A, B, and C in both training and validation cohorts (Figure 3). Altogether, these data underlined the interest of GEP-based DNA repair deregulations, alone or in combination with *NPM1* and *FLT3* mutational status to identify high-risk CN-AML patients.

## 3. Discussion

Despite an improvement in prognosis classification, mostly based on the identification of gene mutations such as *NPM1*, *FLT3,* or *CEBPA*, outcomes in CN-AML remain heterogeneous, underlying the wide diversity of this AML subset. In this study, we developed a GE-based score using data from genes involved in the DNA damage response. Our model succeeded to predict poor outcomes in three independent cohorts of adult patients with CN-AML treated with intensive chemotherapy. Combining DNA repair score with *NPM1* and *FLT3*-ITD mutational status allows us to distinguish three prognostic groups including a low-risk group with a not reached median OS after a median follow-up of more than 3 years in both cohorts, a high-risk group with a median OS of about 8 months in both cohorts, and an intermediate risk-group. Furthermore, the DNA repair score is also a prognostic factor for EFS underlining a potential role of DNA repair pathway deregulation in disease relapse. This model may therefore be used for risk stratification in CN-AML since DNA repair scores could be calculated for new patients using Affymetrix microarray data if the same methodology of normalization is applied. Prospective validation of DNA repair score prognostic value using RNA-seq data will be of interest in future investigations.

Among the GEP-based defined DNA-repair scores built into our study, HRR and NER scores remained independent prognostic factors in CN-AML. HRR pathway is a process involved in DNA double-strand break (DSB) repair, in which complementary sister chromatid is used as a template for an error-free repair of DNA sequence [39,40]. Among the prognostic factors composing the DNA repair score, MRE11A is a nuclease involved in the MRN complex (for MRE11, RAD50 and NBS1) which acts as a sensor for DSB damage [41,42]. RAD52, BRCA2, XRCC2 are proteins directly involved in the DNA repair process [40,43], and RTEL1 and SRCAP are regulators of HRR [44,45]. NER pathway is involved in the recognition and repair of lesions that disrupt DNA double helices, such as adducts or inter-strand crosslinks (ICL) [46,47]. RAD23A and COPS6 are involved in DNA damage recognition. The recruitment of the DNA incision complex, in which ERCC1, ERCC8, and GTF2H2 are involved, is mediated by XPA [46,48]. XRCC1 and EP300 are respectively involved in DNA final ligation process and NER regulation [49,50]. Several polymorphisms in genes involved in HRR and NER have been correlated with AML onset and outcome. RAD51 is a key protein in the HRR pathway. Its polymorphic variant *RAD51-G135C* has been suggested to be correlated with the onset of therapy-related AML by several case-control studies, even if two meta-analyses seem to dismiss the role of this polymorphism in de novo AML onset [51,52,53,54]. XPD is involved in the NER pathway, and its polymorphism XPD-Lys751Gln has been shown to be a risk factor for AML onset [10,51,52]. One study also suggested that this polymorphism worsens the AML prognosis [55]. These data highlight the role of DNA repair pathways in leukemogenesis and suggest their role in chemotherapy resistance.

Interestingly, when compared using multivariate analysis, the DNA repair score and *NPM1*/*FLT3* mutational status remained statistically associated with the outcome in CN-AML. The poor prognosis of *FLT3*-ITD mutated CN-AML has been demonstrated for a high ITD-to-wild-type allelic ratio (>0.5) [56]. Unfortunately, this ratio was not available in these cohorts, and we approximated that all patients with *FLT3*-ITD would have a worse prognosis compared to the others. Moreover, other prognostic mutations defined in ELN 2017 classification (*CEBPA*, *TP53*, *RUNX1*, *ASXL1*, and biallelic mutation of *CEBPA*) were not available. A combination of DNA repair scores with the mutational status for these genes may provide a more precise risk classification of patients according to recommendations. Studying the correlation between our DNA repair score and others frequently mutated genes in AML may also be of interest, especially for genes involved in chromatin organization and stability (*DNMT3A*, *IDH1*/*2*, *TET2*, *KMT2A*, *EZH2*). Therefore, it will be compelling to investigate the link between *FLT3*-ITD allelic ratio, recurrent CN-AML mutations, and the described GEP-based DNA-repair risk score in a new prospective cohort.

Intensive chemotherapy for CN-AML patients usually includes cytarabine and anthracyclines (daunorubicine or idarubicine) [57]. Cytarabine, a nucleoside analog, incorporates into DNA and interferes with DNA synthesis during the phase S of the cell cycle, leading to genomic instability [58]. Anthracyclines are DNA topoisomerase II inhibitors that induce DNA damages such as DSB, adducts, and ICL [58]. Therefore, overexpression of HRR or NER pathway genes could be associated with chemotherapy resistance, but a better understanding of the functional role of DNA repair pathways in the pathogenesis and drug resistance of CN-AML is needed [59]. Gene silencing approaches by sh-RNA or CRISPR-Cas9 strategies could be of particular interest. Thus, CRISPR-Cas9 or RNAi screening revealed that *APEX1* (BER), *RTEL1* (HRR), and *COPS6* (NER) are essential AML genes. Among these genes, COPS6 overexpression is associated with poor outcomes in many solid tumors. Interestingly, COPS6 depletion showed in vivo efficacy against glioblastoma [60], cervical cancer [61], or papillary thyroid carcinoma [62], through the regulation of several signaling pathways. However, the biological function of COPS6 in leukemogenesis and AML drug-resistance remains largely unknown.

Therefore, inhibiting DNA repair might be a promising strategy to improve the efficacy of genotoxic drugs and overcome drug resistance, according to the principle of “synthetic lethality” [63,64]. Poly-(ADP Ribose) Polymerase (PARP) inhibitors (talazoparib, olaparib) has been recently approved for the treatment of breast and ovarian cancers with BRCA1/2 mutations, and several others drugs inhibiting or modulating DNA repair pathways are currently in clinical development for cancer therapy (Table 5). In preclinical studies, APEX1 inhibitor has demonstrated promising toxicity on primary AML cells in vitro, alone or in association with hypomethylating agent decitabine or with PARP inhibitor talazoparib. Even if APEX1 expression levels did not significantly differ between responding and non-responding AML cells, APEX1 inhibitor appeared promising in normal karyotype AML (83% of the patients with response to APEX1 inhibitor) [65]. Moreover, several other compounds targeting proteins involved in DNA repair mechanisms have already shown promising results as potential new anti-cancer drugs in pre-clinical studies, such as BLM [66], WRN [67], RAD52 [68], or MRE11 [69] inhibitors.

Our data support the potential of DNA repair scores to identify CN-AML patients whose malignant cells are dependent on specific DNA repair pathways to design targeted therapy with ATM, CHEK-1/2, RAD51, or CDK7 inhibitors exploiting the addiction to deregulated DNA repair mechanisms.

## 4. Materials and Methods

### 4.1. Patients and Gene Expression Data

Gene expression microarray data from two independent cohorts of adult patients diagnosed with CN-AML were used. One patient was excluded from each cohort due to a diagnosis of myelodysplastic syndrome. Thus, the first cohort (training set) included 162 patients and the second one (validation set) 78 patients. At least 20 metaphases were analyzed for each patient to confirm the normal karyotype. At the beginning of treatment, the median age was 57.5 years in the training cohort and 62 years in the validation cohort. Pretreatment clinical characteristics of patients have been described previously [70]. At diagnosis, training and validation cohorts statistically differed according to FLT3-ITD positive patients (48% vs. 22%, respectively) and median leucocyte count (36.9 G/L vs. 15.9 G/L, respectively). *NPM1* and *FLT3* mutational status were kindly provided for each patient by Metzeler et al. [70]. *FLT3*-ITD allelic ratio and mutational status for other prognostic genes (*CEBPA*, *TP53*, *ASXL1*, *RUNX1*) were not available in these cohorts. All patients were treated with intensive chemotherapy. Another cohort (Verhaak cohort) comprising 181 patients with de novo CN-AML was used for validation. The median age at diagnosis was 46 years in this cohort All the patient’s characteristics have been previously reported [36,37,38].

Affymetrix gene expression data are publicly available via the online Gene Expression Omnibus (http://www.ncbi.nlm.nih.gov/geo/) under accession number GSE12417 and GSE14468. They were performed using Affymetrix HG-U133 A&B arrays for training cohort and Affymetrix HG-U133 Plus 2.0 arrays for the validation and Verhaak sets. Normalization of microarray data was performed using the variance stabilizing normalization algorithm, and probe set signals calculated by the median polish method [36,37,38,70,71]. Quality control consisted of visual inspection of the array image for artifacts, assessment of RNA degradation plots, and inspection of rank-vs-residual plots after normalization and probe set summarization. 

### 4.2. Selection of Prognostic Genes

DNA repair gene list was defined using the REPAIRtoire database (http://repairtoire.genesilico.pl) and review of the literature (Table S1) [33]. To establish gene expression (GE)-based risk scores, we selected probe sets whose expression values were significantly associated with overall survival using MaxStat R function, which allows us to determine the optimal cutpoint for continuous variables, and Benjamini–Hochberg multiple testing correction in two independent cohorts (adjusted *p*-value < 0.05).

### 4.3. Building DNA Repair Gene Expression-Based Risk Score

For each pathway, a GE-based risk score was created as the sum of the beta coefficients weighted by +1 or −1 according to the patient signal above or below/equal the probe set MaxStat value as previously reported [32,33,34,35]. Patients from the training cohort were ranked according to increased prognostic score and for a given score value X, the difference in survival of patients with a prognostic score ≤X or >X was computed using MaxStat analysis.

Cox proportional hazards model was performed to determine statistically significant pathway scores in multivariate analysis. A global DNA repair score was calculated based on the pathway scores which remained statistically significant in this analysis. Survival analyses were assessed using the Kaplan–Meier method, and survival curves were compared using the log-rank test.

### 4.4. Validation of the DNA Repair Score on Validation Cohort

Pathway and DNA repair scores were individually calculated in both validation and Verhaak cohort, using the cutoff values determined for the training cohort. Survival analyses were assessed using Kaplan–Meier method, and survival curves were compared using the log-rank test.

### 4.5. Statistical Analyses

All statistical tests were two-tails and Alpha-risk was fixed at 5%. Analyses were performed using R.3.6.0. (R Foundation for Statistical Computing, Vienna, Austria) and SPSS Statistics version 23.0.0.0 for Mac (SPSS Inc., Chicago, IL, USA).

## 5. Conclusions

The DNA repair score may be useful to identify high-risk CN-AML patients and define the best DNA repair inhibitor to use in combination with conventional treatment to improve patients’ outcomes. The DNA repair score could also be valuable for adapting targeted treatment according to the drug resistance mechanisms selected during the clonal evolution of relapsing AML. These advances may improve the survival of CN-AML patients, and limit the side effects of treatment, improving compliance with dosing regimens and overall quality of life.

## Figures and Tables

**Table 1:** List of the 23 probe sets associated with good or bad prognosis in cytogenetically normal acute myeloid leukemias (CN-AML). Corresponding DNA repair pathway, gene symbol, adjusted p-value, hazard ratio, and prognosis significance are provided for each gene.

DNA Repair Pathway	Probe Set	Gene Symbol	Benjamini–Hochberg Corrected *p*-Value	Hazard Ratio	Prognosis
**Base Excision** **Repair pathway** **(BER)**	210027_s_at 209731_at 202330_s_at 203655_at	APEX1 NTHL1 UNG XRCC1	0.02 0.0016 0.0095 0.022	1.6 1.9 2 1.6	Bad Bad Bad Bad
**Fanconi** **pathway** **(FANC)**	209902_at 214727_at 203719_at 203678_at 221206_at 219317_at	ATR BRCA2 ERCC1 FAN1 PMS2 /// PMS2CL POLI	0.0048 0.0049 0.0037 0.0028 0.024 0.0016	1.8 0.58 1.9 1.8 1.8 1.9	Bad Good Bad Bad Bad Bad
**Homologous Recombination Repair** **pathway** **(HRR)**	214727_at 205395_s_at 205647_at 206092_x_at 212275_s_at 207598_x_at	BRCA2 MRE11A RAD52 RTEL1 SRCAP XRCC2	0.0049 0.015 0.044 0.00047 0.014 0.007	0.58 1.8 1.9 2.5 0.6 1.7	Good Bad Bad Bad Good Bad
**Mismatch Repair pathway** **(MMR)**	205887_x_at 221206_at 1053_at	MSH3 PMS2 /// PMS2CL RFC2	0.000043 0.024 0.023	2.8 1.8 1.6	Bad Bad Bad
**Nucleotide Excision** **Repair** **pathway** **(NER)**	201405_s_at 213579_s_at 203719_at 205162_at 223758_s_at 201046_s_at 205672_at 203655_at	COPS6 EP300 ERCC1 ERCC8 GTF2H2 RAD23A XPA XRCC1	0.011 0.019 0.0037 0.04 0.033 0.0067 0.0035 0.022	1.7 0.59 1.9 1.5 1.5 0.53 1.8 1.6	Bad Good Bad Bad Bad Good Bad Bad

**Table 2:** Cox analysis of overall survival in CN-AML training cohort (*n* = 162) according to DNA repair pathway scores. Hazard ratio (HR) and p-values are shown for each DNA repair pathway score in univariate and multivariate Cox analysis. NS: not significant.

DNA Repair Pathway Score	Univariate Cox Analysis		Multivariate Cox Analysis	
	HR	*p*-Value	HR	*p*-Value
BER score	1.97	1.44 × 10^−3^	0.93	NS
FANC score	2.32	2.98 × 10^−5^	1.30	NS
HRR score	3.23	2.16 × 10^−7^	2.36	5.89 × 10^−4^
MMR score	2.80	1.59 × 10^−4^	1.58	NS
NER score	3.83	2.90 × 10^−4^	2.54	1.66 × 10^−2^

**Table 3:** Cox analysis of overall survival in CN-AML training cohort (*n* = 162) according to DNA repair score, and *NPM1*/*FLT3* mutational status. Hazard ratio (HR) and p-values are shown for each parameter in univariate and multivariate Cox analysis. ITD: internal tandem duplication.

Scores	Univariate Cox Analysis		Multivariate Cox Analysis	
	HR	*p*-Value	HR	*p*-Value
**DNA repair score**	2.76	1.49 × 10^−8^	2.66	5.1 × 10^−8^
***NPM1* mutation/*FLT3*-ITD classification**	1.81	1.18 × 10^−4^	1.76	6.2 × 10^−4^

**Table 4:** DNA repair score and NPM1/FLT3 mutational status combination to establish a global prognosis score in CN-AML. Patients were classified according to DNA repair score risk group (I, II or III) and NPM1/FLT3 mutational status.

		Classification According to DNA Repair Score		
		Group I 0 point	Group II 1 point	Group III 2 points
***NPM1*** **and** ***FLT3*** **mutational** **status**	*NPM1*+ and *FLT3*-ITD- 0 point	**0**	**1**	**2**
	*NPM1*+ and *FLT3*-ITD+ *or* *NPM1*- and *FLT3*-ITD- 1 point	**1**	**2**	**3**
	*NPM1*- and *FLT3*-ITD+ 2 points	**2**	**3**	**4**

Patients with NPM1 mutation or FLT3-ITD are respectively designated by NPM1+ and FLT3-ITD+. Patients without NPM1 mutation or FLT3-ITD are respectively designated by NPM1- and FLT3-ITD-. Points were attributed as described in the table. Patients with 0 or 1 point were grouped in group A (green), patients with 2 points were grouped in group B (yellow), and patients with 3 or 4 points were grouped in group C (red). ITD: internal tandem duplication.

**Table 5:** A non-exhaustive list of recruiting clinical trials for DNA repair targeting drugs in 2020 (www.clinicaltrials.gov). ADK: adenocarcinoma. AML: acute myeloid leukemia. ASCT: autologous stem cell transplant. GO: gemtuzumab ozogamicin. MDS: myelodysplastic syndrome. R/R: relapsed or refractory. SQCLC: squamous cell lung carcinoma.

Target	Drug	Cancer	Phase	Intervention	Identifier
Base Excision Repair (BER) Pathway					
**APEX1**	TRC-102	Solid tumors & lymphomas	I/II	TRC-102 + temozolomide	NCT01851369
**PARP1/2**	Niraparib	Pancreatic ADK	II	Niraparib alone	NCT03601923
	Olaparib	Lymphomas (B/T/Hodgkin)	I	Olaparib + high-dose chemotherapy + ASCT	NCT03259503
	Olaparib	AML or MDS with *IDH*1/2 mutation	II	Olaparib alone	NCT03953898
	Talazoparib	R/R AML CD33+	I/II	Talazoparib + GO	NCT04207190
	Talazoparib	AML	I/II	Talazoparib + Decitabine	NCT02878785
	Veliparib	Myeloproliferative disorders	II	Carboplatin + Topotecan +/− Veliparib	NCT03289910
**Homologous Recombination Repair (HRR) Pathway**					
**ATM**	AZD1390	Glioblastoma	I	AZD1390 + radiotherapy	NCT03423628
**CHEK-1/2**	Prexasertib	R/R medulloblastoma	I	Prexasertib + Gemcitabine *or* Cyclophosphamide	NCT04023669
**RAD51**	CYT-0851	Solid tumors & B-cell lymphomas	I/II	CYT-0851 alone	NCT03997968
**Fanconi (FANC) Pathway**					
**ATM**	(see above)				
**ATR**	Ceralasertib	R/R non-Hodgkin’s lymphoma	I	Ceralasertib + Acalabrutinib	NCT03527147
	M6620	Solid tumors	II	MS6620 alone	NCT03718091
**RAD51**	(see above)				
**Nucleotide Excision Repair (NER) pathway**					
**CDK7**	LY3405105	Solid tumors	I	LY3405105 alone	NCT03770494
	SY5609	Solid tumors	I	SY5609 +/− Fulvestrant	NCT04247126
	CT7001	Solid tumors	I/II	CT7001 +/− Fulvestrant	NCT03363893
**Others**					
**WEE1**	Adavosertib	SQCLC	II	Adavosertib + Paclitaxel + Carboplatin	NCT02513563
**PARP1/2 +** **ATR *or*** **WEE1**	Olaparib Ceralasertib Adavosertib	Metastatic triple negative breast cancer	II	Olaparib alone *or* Olaparib + Ceralasertib *or* Olaparib + Adavosertib	NCT03330847

## Supplementary Materials

The following are available online at https://www.mdpi.com/2072-6694/12/10/2874/s1, Figure S1: Prognostic value of DNA repair pathway scores in CN-AML patients of the training cohort, Figure S2: Kaplan-Meier survival curves for training and validation cohorts, Figure S3: Kaplan-Meier survival curves according to risk stratification determined by DNA repair score in Verhaak cohort, Figure S4: Distribution of age (years) in each DNA repair score subgroups (I, II and III) for training and validation cohorts, Figure S5: Kaplan-Meier survival curves according to *NPM1*/*FLT3* mutational status, Figure S6: Kaplan-Meier survival curve according to the points allotted to patients in Table 4 for the training cohort, Table S1: Genes coding for proteins involved in DNA repair, Table S2: Cox analysis of overall survival in CN-AML validation cohort according to DNA repair pathway scores, Table S3: Cox analysis of overall survival in CN-AML validation cohort according to DNA repair score, and *NPM1* & *FLT3* mutational status.
