# Supplementary material for: DNA Repair Expression Profiling to Identify High-Risk Cytogenetically Normal Acute Myeloid Leukemia and Define New Therapeutic Targets
Source: Cancers (Basel). 2020 Oct 6;12(10):2874. doi: 10.3390/cancers12102874 (PMC7599826; doi:10.3390/cancers12102874)
Supplement: Supplementary file 1 [file cancers-12-02874-s001.pdf]

cancers

MDPI

Supplementary Materials

DNA Repair Expression Profiling to Identify High-Risk Cytogenetically Normal Acute Myeloid Leukemia and Define New Therapeutic Targets

Table S1. Genes coding for proteins involved in DNA repair. Gene symbols are provided with corresponding probe sets for each DNA repair pathway.

**Base Excision Repair (BER) pathway**					
210027_s_at	APEX1	226585_at	NEIL2	212836_at	POLD3
204408_at	APEX2	219502_at	NEIL3	202996_at	POLD4
218527_at	APTX	209731_at	NTHL1	216026_s_at	POLE
204767_s_at	FEN1	205301_s_at	OGG1	233852_at	POLH
204883_s_at	HUS1	208644_at	PARP1	221049_s_at	POLL
202726_at	LIG1	215773_x_at	PARP2	218685_s_at	SMUG1
207348_s_at	LIG3	201202_at	PCNA	203743_s_at	TDG
214048_at	MBD4	218961_s_at	PNKP	202330_s_at	UNG
203686_at	MPG	203616_at	POLB	203655_at	XRCC1
207727_s_at	MUTYH	203422_at	POLD1		
219396_s_at	NEIL1	201115_at	POLD2		

**Fanconi (FANC) pathway**					
213454_at	APITD1	1557217_a_at	FANCB	205024_s_at	RAD51
208442_s_at	ATM	205189_s_at	FANCC	206066_s_at	RAD51C
209902_at	ATR	223545_at	FANCD2	218428_s_at	REV1
1552937_s_at	ATRIP	220255_at	FANCE	218979_at	RMI1
205733_at	BLM	222713_s_at	FANCF	226456_at	RMI2
204531_s_at	BRCA1	203564_at	FANCG	201529_s_at	RPA1
214727_at	BRCA2	213008_at	FANCI	201756_at	RPA2
221800_s_at	C17orf70	218397_at	FANCL	209507_at	RPA3
214816_x_at	C19orf40	242711_x_at	FANCM	218317_x_at	SLX1
205394_at	CHEK1	202520_s_at	MLH1	233334_x_at	SLX1A
203229_s_at	CLK2	218463_s_at	MUS81	239687_at	SLX4
234464_s_at	EME1	219530_at	PALB2	214299_at	TOP3A
203719_at	ERCC1	221206_at	PMS2	202633_at	TOPBP1
228131_at	ERCC1/ASE1	209805_at	PMS2///PMS2CL	202412_s_at	USP1
235215_at	ERCC4	233852_at	POLH	65591_at	WDR48
203678_at	FAN1	219317_at	POLI		
203805_s_at	FANCA	242804_at	POLN		

**Homologous Recombination Repair (HRR) pathway**					
208442_s_at	ATM	227286_at	INO80E	204146_at	RAD51AP1
205345_at	BARD1	214258_x_at	KAT5	210255_at	RAD51B
205733_at	BLM	202726_at	LIG1	206066_s_at	RAD51C
204531_s_at	BRCA1	224320_s_at	MCM8	37793_r_at	RAD51D
214727_at	BRCA2	219673_at	MCM9	205647_at	RAD52
214816_x_at	C19orf40	205395_s_at	MRE11A	219494_at	RAD54B
210416_s_at	CHEK2	210533_at	MSH4	203344_s_at	RBBP8
208386_x_at	DMC1	210410_s_at	MSH5///MSH5-SAPCD1///SAPCD1	221686_s_at	RECQL5
234464_s_at	EME1			201529_s_at	RPA1
1569868_s_at	EME2	218463_s_at	MUS81	201756_at	RPA2
204603_at	EXO1	202907_s_at	NBN	209507_at	RPA3
224683_at	FBXO18	219530_at	PALB2	206092_x_at	RTEL1
228286_at	GEN1	203422_at	POLD1	212275_s_at	SRCAP

225357_s_at	INO80	201115_at	POLD2	214299_at	TOP3A
65133_i_at	INO80B///INO80B-WBP1	212836_at	POLD3	207598_x_at	XRCC2
		202996_at	POLD4	216299_s_at	XRCC3
1559716_at	INO80C	208393_s_at	RAD50		
227931_at	INO80D	205024_s_at	RAD51		
**Mismatch Repair (MMR) pathway**					
204603_at	EXO1	1554742_at	PMS1	208021_s_at	RFC1
202726_at	LIG1	221206_at	PMS2	1053_at	RFC2
202520_s_at	MLH1	209805_at	PMS2///PMS2CL	204127_at	RFC3
204838_s_at	MLH3	203422_at	POLD1	204023_at	RFC4
209421_at	MSH2	201115_at	POLD2	203209_at	RFC5
205887_x_at	MSH3	212836_at	POLD3	201529_s_at	RPA1
202911_at	MSH6	202996_at	POLD4	209507_at	RPA3
201202_at	PCNA	216026_s_at	POLE		
**Nucleotide Excision Repair (NER) pathway**					
204093_at	CCNH	235215_at	ERCC4	216026_s_at	POLE
211297_s_at	CDK7	202414_at	ERCC5	202725_at	POLR2A
209194_at	CETN2	207347_at	ERCC6	201046_s_at	RAD23A
202467_s_at	COPS2	205162_at	ERCC8	201222_s_at	RAD23B
202078_at	COPS3	202451_at	GTF2H1	218117_at	RBX1
218042_at	COPS4	223758_s_at	GTF2H2	208021_s_at	RFC1
201652_at	COPS5	222104_x_at	GTF2H3	201529_s_at	RPA1
201405_s_at	COPS6	203577_at	GTF2H4	201756_at	RPA2
209029_at	COPS7A	213357_at	GTF2H5	209507_at	RPA3
219997_s_at	COPS7B	200943_at	HMGN1	216241_S_at	TCEA1
236204_at	COPS8	202726_at	LIG1	203919_at	TCEA2
201423_s_at	CUL4A	207348_s_at	LIG3	226388_at	TCEA3
208619_at	DDB1	202167_s_at	MMS19	233893_s_at	UVSSA
203409_at	DDB2	203565_s_at	MNAT1	218110_at	XAB2
213579_s_at	EP300	201202_at	PCNA	205672_at	XPA
203719_at	ERCC1	203422_at	POLD1	209375_at	XPC
228131_at	ERCC1/ASE1	201115_at	POLD2	203655_at	XRCC1
213468_at	ERCC2	212836_at	POLD3		
202176_at	ERCC3	202996_at	POLD4		
**Non-Homologous End Joining (NHEJ) pathway**					
241379_at	APLF	209940_at	PARP3	1569098_s_at	TP53BP1
208442_s_at	ATM	218961_s_at	PNKP	205667_at	WRN
235478_at	DCLRE1C	221049_s_at	POLL	205072_s_at	XRCC4
205436_s_at	H2AFX	222238_s_at	POLM	232633_at	XRCC5
206235_at	LIG4	210543_s_at	PRKDC	200792_at	XRCC6
219418_at	NHEJ1	206554_x_at	SETMAR		
210470_x_at	NONO	201585_s_at	SFPQ		

Table S2. Cox analysis of overall survival in CN-AML validation cohort (n = 78) according to DNA repair pathway scores. Hazard ratio (HR) and p-values are shown for each HRR and NER repair pathway scores (computed with training cohort parameters) in univariate Cox analysis.

DNA repair pathway score	Univariate Cox analysis	
	**HR**	**p-value**
**HRR score**	3.73	1.32×10^{-5}
**NER score**	2.83	0.028

Table S3. Cox analysis of overall survival in CN-AML validation cohort (n = 78) according to DNA repair score, and NPM1 & FLT3 mutational status. Hazard ratio (HR) and p-values are shown for each parameter in univariate and multivariate Cox analysis. NS: not significant. ITD: internal tandem duplication.

Scores	Univariate Cox analysis		Multivariate Cox analysis	
	**HR**	***p*-value**	**HR**	***p*-value**
**DNA repair score**	3.04	1.01×10^{-5}	3.07	1.4×10^{-5}
***NPM1* mutation / *FLT3*-ITD classification**	1.71	0.020	1.67	0.027

(**a**)

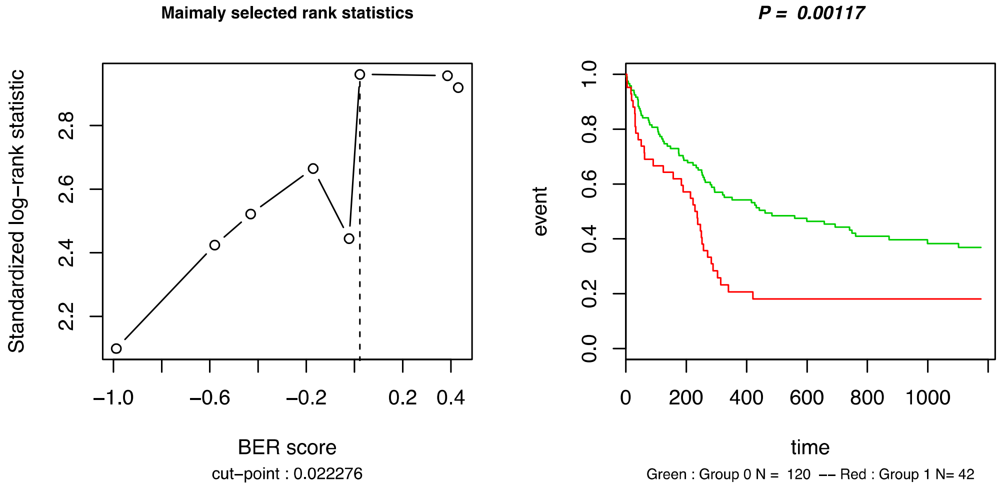
Maimaly selected rank statistics
Standardized log−rank statistic
2.2
2.4
2.6
2.8
−1.0
−0.6
−0.2
0.2
0.4
BER score
cut−point : 0.022276
P = 0.00117
event
0.0
0.2
0.4
0.6
0.8
1.0
0
200
400
600
800
1000
time
Green : Group 0 N = 120 −− Red : Group 1 N= 42

(**b**)

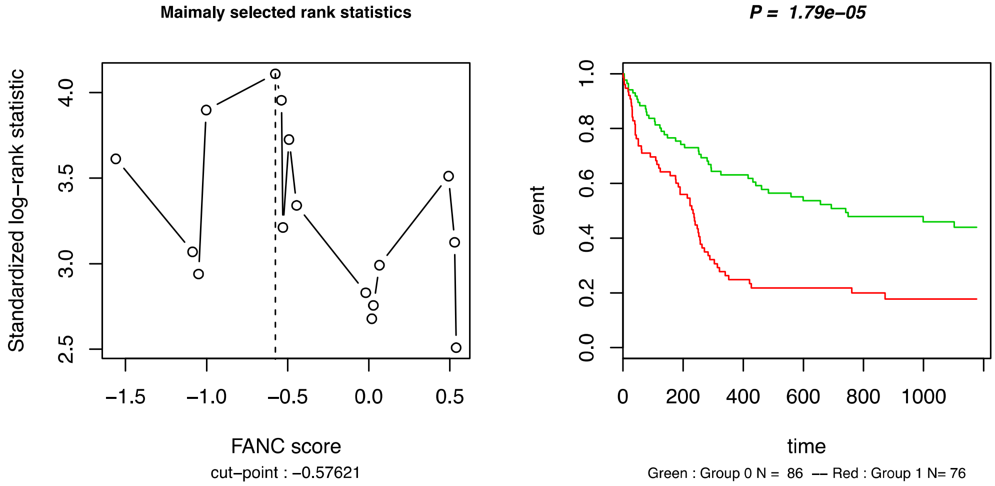
Maimaly selected rank statistics
Standardized log−rank statistic
2.5
3.0
3.5
4.0
−1.5
−1.0
−0.5
0.0
0.5
FANC score
cut−point : −0.57621
P = 1.79e−05
event
0.0
0.2
0.4
0.6
0.8
1.0
0
200
400
600
800
1000
time
Green : Group 0 N = 86 −− Red : Group 1 N= 76

(**c**)

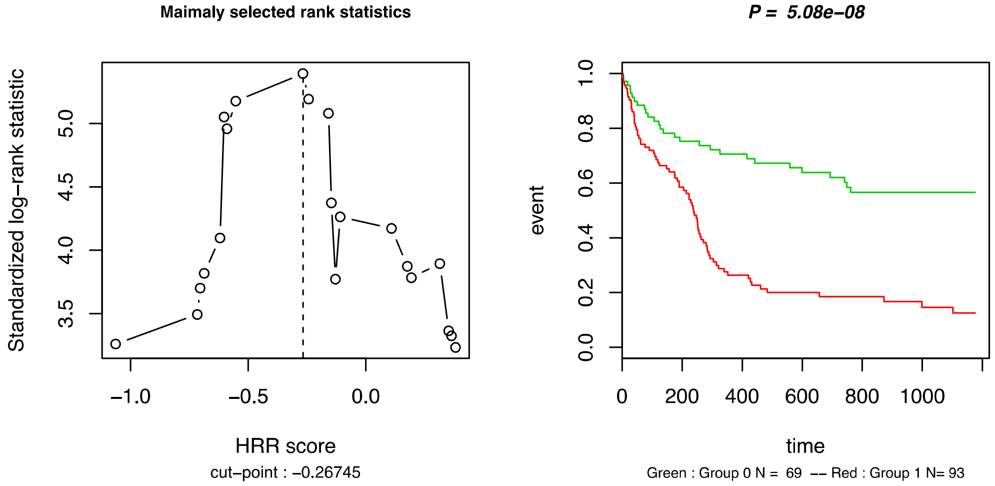
Maimaly selected rank statistics
Standardized log−rank statistic
3.5
4.0
4.5
5.0
−1.0
−0.5
0.0
HRR score
cut−point : −0.26745
P = 5.08e−08
event
0.0
0.2
0.4
0.6
0.8
1.0
0
200
400
600
800
1000
time
Green : Group 0 N = 69 −− Red : Group 1 N= 93

(**d**)

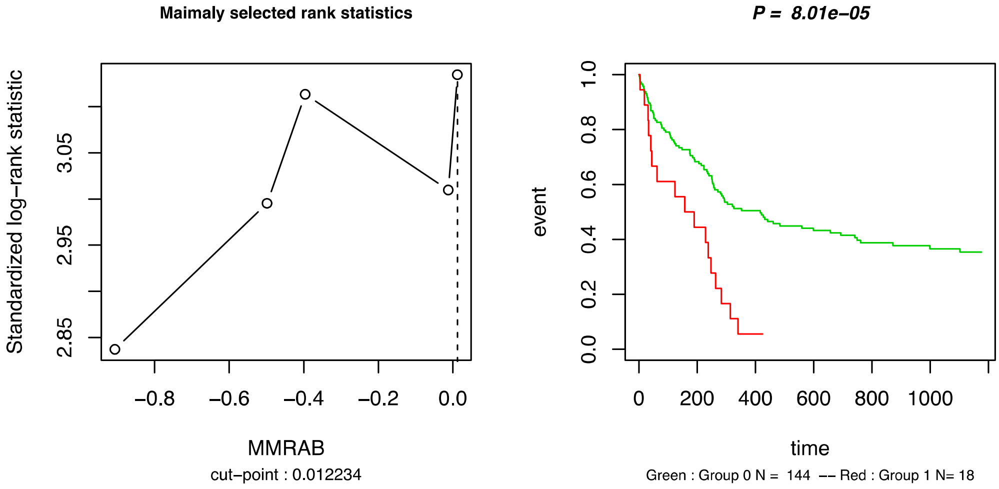

(**e**)

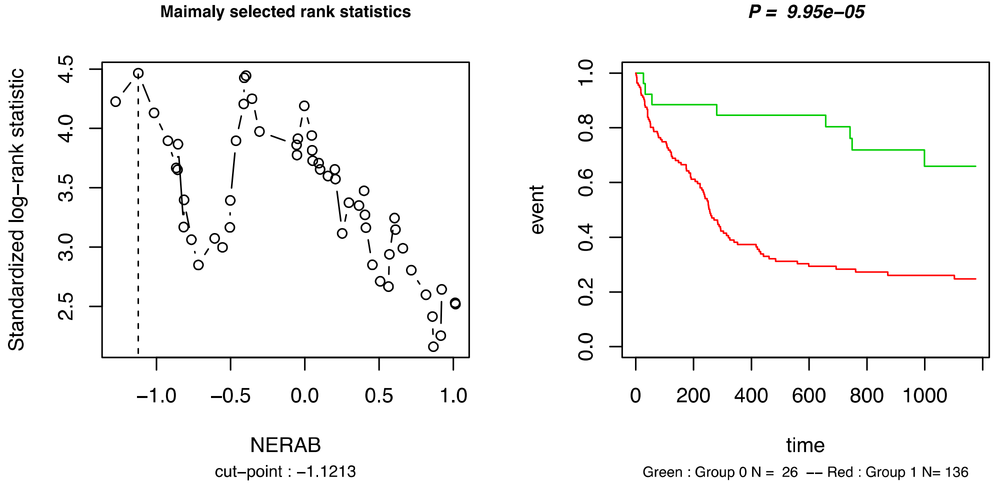

Figure S1. Prognostic value of DNA repair pathway scores in CN-AML patients of the training cohort. Patients of the training cohort (n = 162) were ranked according to increasing BER (**a**), FANC (**b**), HRR (**c**), MMR (**d**) and NER (**e**) scores and a maximum difference in OS was obtained using MaxStat R function. Green survival curves represent patients whose score is inferior or equal to the MaxStat determined cut-point. Red survival curves designate patients whose score is strictly superior to the MaxStat determined cut-point.

(**a**)

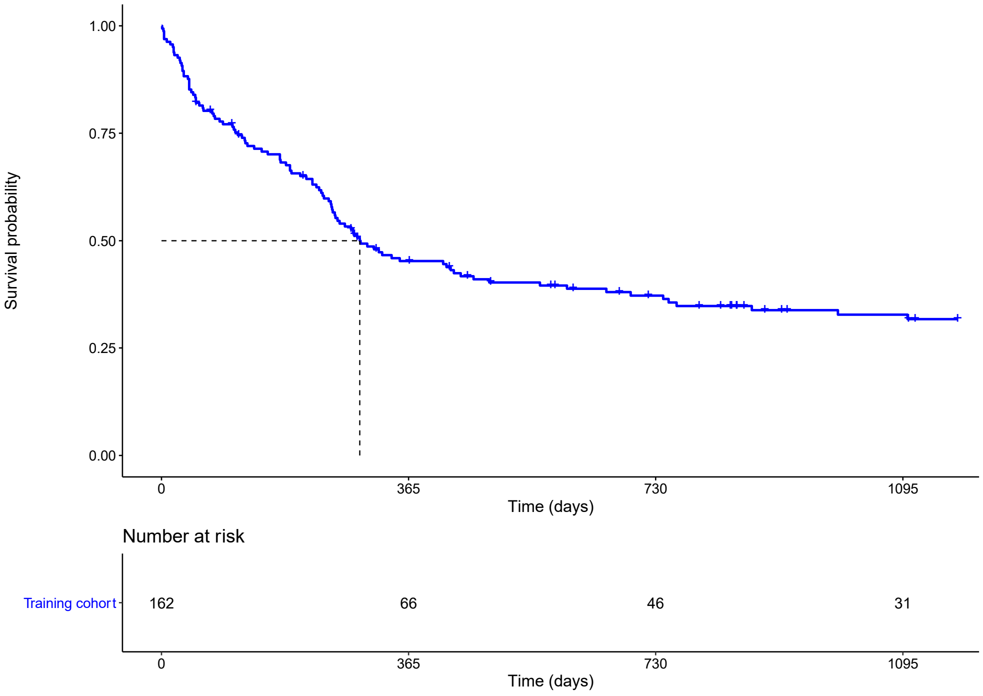

Survival probability
1.00
0.75
0.50
0.25
0.00
0
365
730
1095
Time (days)
Number at risk
Training cohort
162
66
46
31
0
365
730
1095
Time (days)

(**b**)

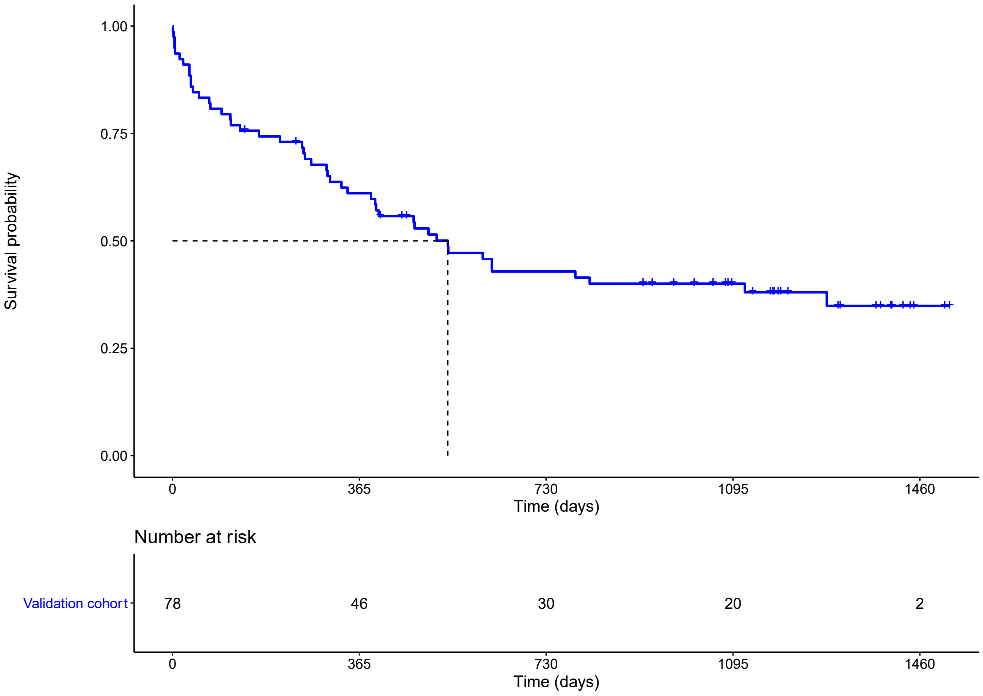

Survival probability
1.00
0.75
0.50
0.25
0.00
0
365
730
1095
1460
Time (days)
Number at risk
Validation cohort
78
46
30
20
2
0
365
730
1095
1460
Time (days)

(**c**)

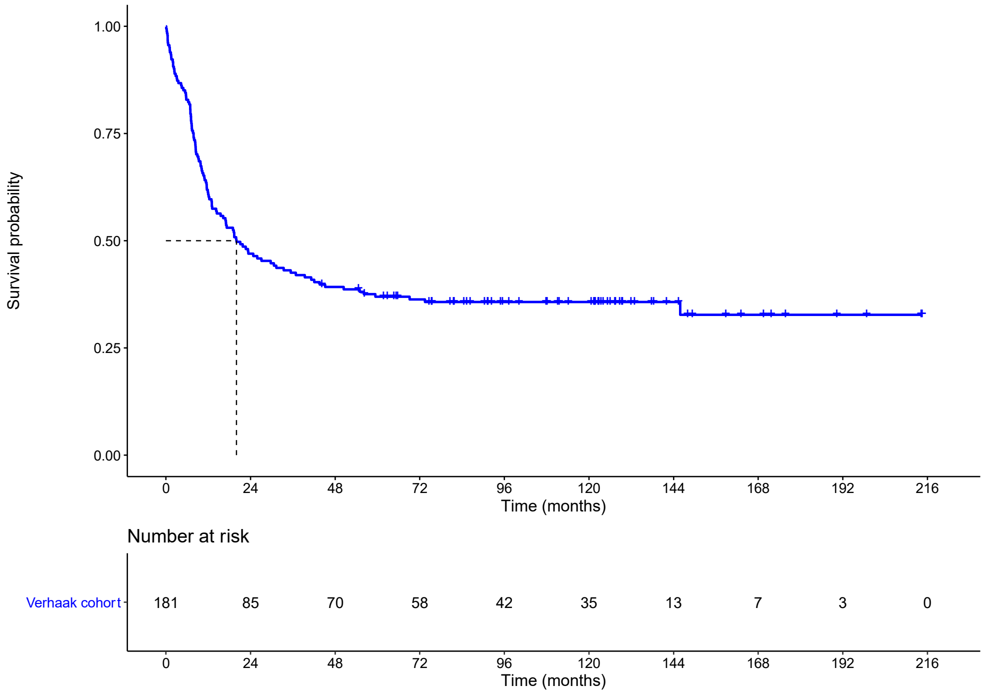

Figure S2. Kaplan-Meier survival curves for training and validation cohorts. (**a**) Kaplan-Meier survival curve for the whole training cohort (*n* = 162). After a median follow-up of 1176 days (95% CI: 916-NR), median overall survival (OS) was 293 days (95% CI: 252–461) for the whole training cohort. (**b**) Kaplan-Meier survival curve for the whole validation cohort (*n* = 78). After a median follow-up of 1183 days (95% CI: 1092–1383), median overall survival (OS) was 538 days (95% CI: 388–1278) for the whole validation cohort. (**c**) Kaplan-Meier survival curve for the whole Verhaak cohort (*n* = 181). After a median follow-up of 122 months (95% CI: 108–128), median overall survival (OS) was 20 months (95% CI: 14.4–36.8) for the whole Verhaak cohort. NR: not reached.

(**a**)

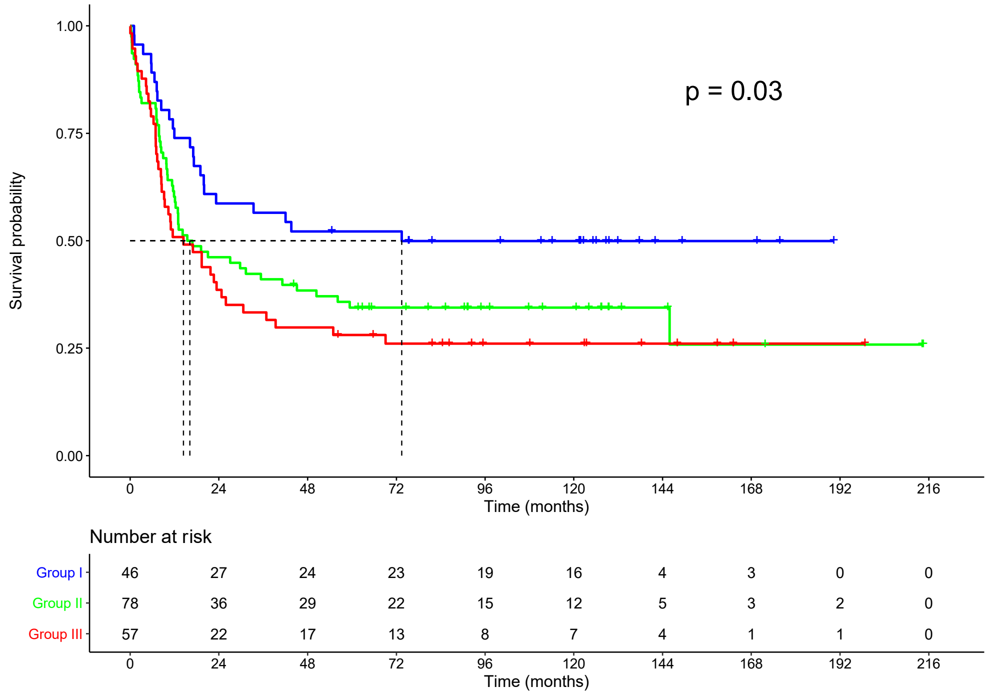

(**b**)

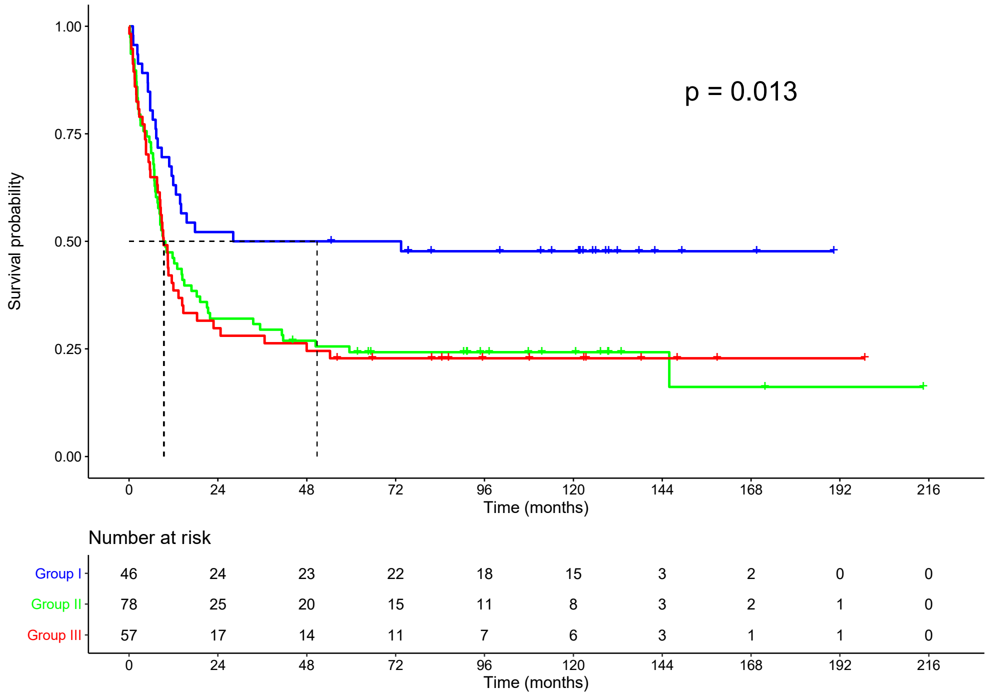

Figure S3. Kaplan-Meier survival curves according to risk stratification determined by DNA repair score in Verhaak cohort (n = 181). (**a**) Kaplan-Meier survival curve for overall survival. Median OS was 73.5 months (95% CI: 20.0-NR), 16.2 months (95% CI: 12.1–50.4) and 14.4 months (95% CI: 8.5–25.9) respectively for patients in groups I (low DNA repair score), II (medium DNA repair score) and III (high DNA repair score). One-year OS was 73.9% (95% CI: 62.3-87.8) in group I, 60.3% (95% CI: 50.3–72.1) in group II, and 50.9% (95% CI: 39.4–65.7) in group III. (**b**) Kaplan-Meier survival curve for event-free survival. Median EFS was 50.8 months (95% CI: 12.7-NR), 9.5 months (95% CI: 7.69-NR) and 9.3 months (95% CI: 7.8-NR) respectively for patients in groups I, II and III. One-year EFS was 63.0% (95% CI: 50.5–78.7) in group I, 46.2% (95% CI: 36.3–58.7) in group II, and 38.6% (95% CI: 27.8–53.6) in group III. p-values were determined with log-rank test. NR: not reached.

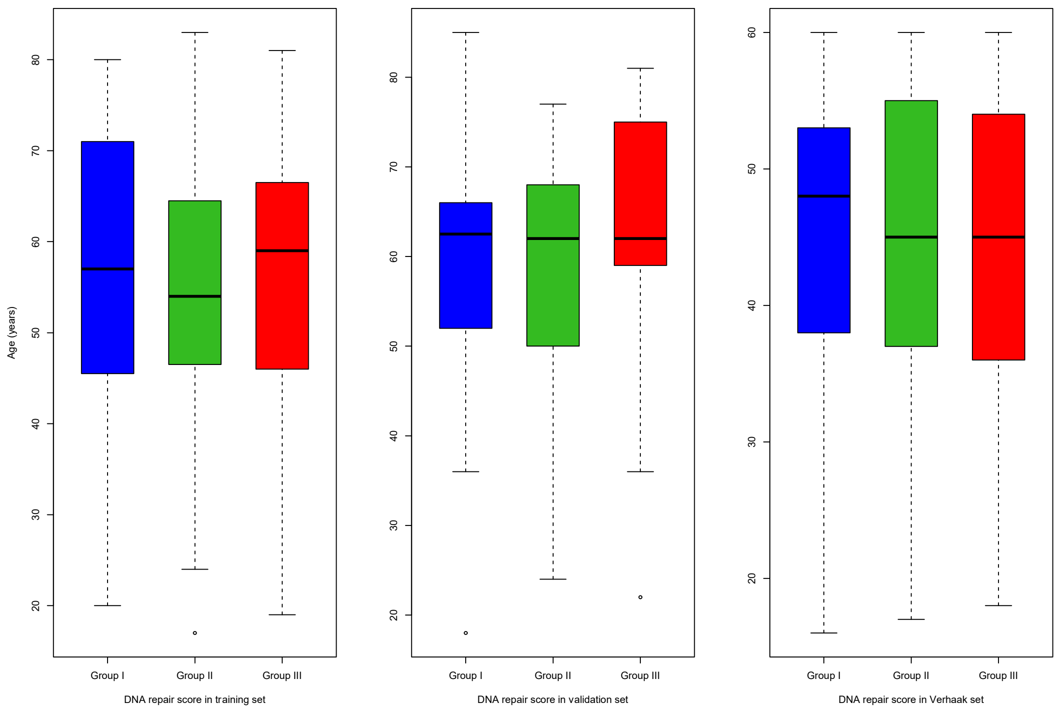

Figure S4. Distribution of age (years) in each DNA repair score subgroups (I, II and III) for training and validation cohorts. Statistical analysis demonstrated no difference between subgroups according to age in training set (median age of 57, 54 and 59 years in respectively Group I, II and III, $p = 0.61$), validation set (median age of 62.5, 62 and 62 years in respectively Group I, II and III, $p = 0.34$) and Verhaak set (median age of 48, 45 and 45 years in respectively Group I, II and III, $p = 0.95$). P-values were estimated with Kruskall-Wallis test.

(**a**)

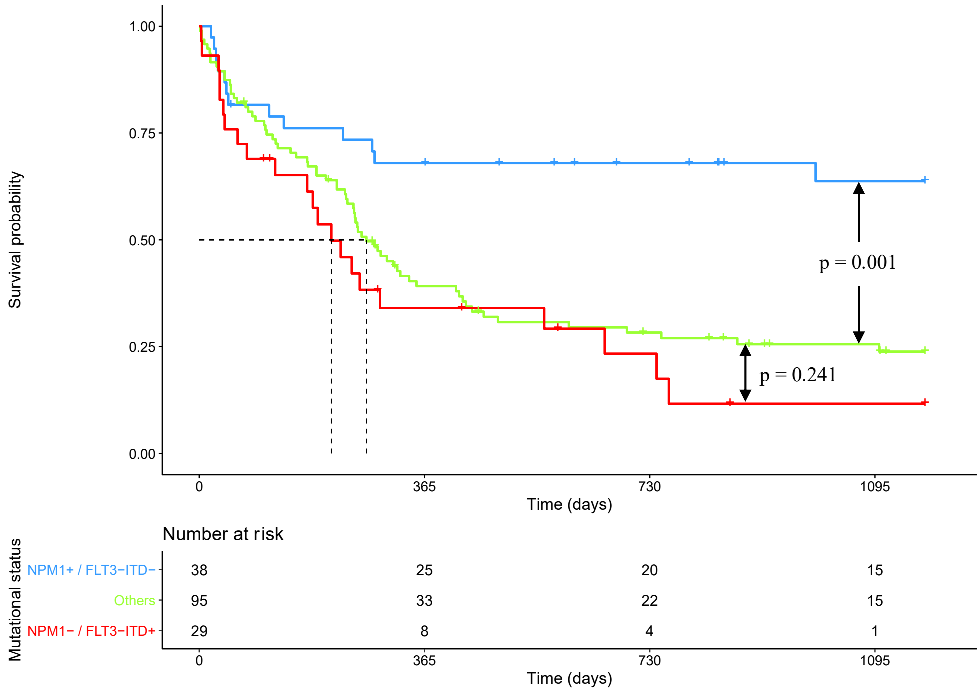

(**b**)

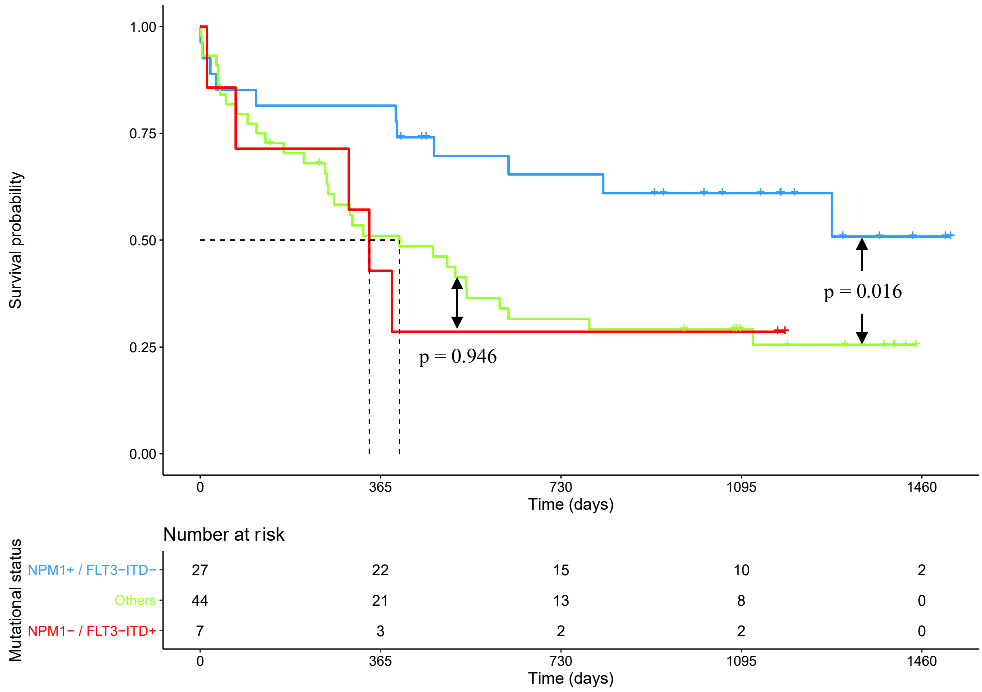

Figure S5. Kaplan-Meier survival curves according to NPM1/FLT3 mutational status. (**a**) Kaplan-Meier survival curve for the training cohort (*n* = 162). Median OS was not reached (95% CI: 999-NR) for patients with NPM1+/FLT3-ITD- mutational status, 271 days (95% CI: 240–416) for patients with NPM1+/FLT3-ITD+ or NPM1-/FLT3- mutational status ("Others") and 214 days (95% CI: 123–657) for patients with NPM1-/ FLT3-ITD+ mutational status. (**b**) Kaplan-Meier survival curve for the validation cohort (*n* = 78). Median OS was not reached (95% CI: 624-NR) for patients with NPM1+/FLT3-ITD- mutational status, 403 days (95% CI: 259–624) for patients with NPM1+/FLT3-ITD+ or NPM1-/FLT3- mutational status ("Others") and 342 days (95% CI: 72-NR) for patients with NPM1-/ FLT3-ITD+ mutational status. P-values were estimated with log-rank test. NR: not reached.

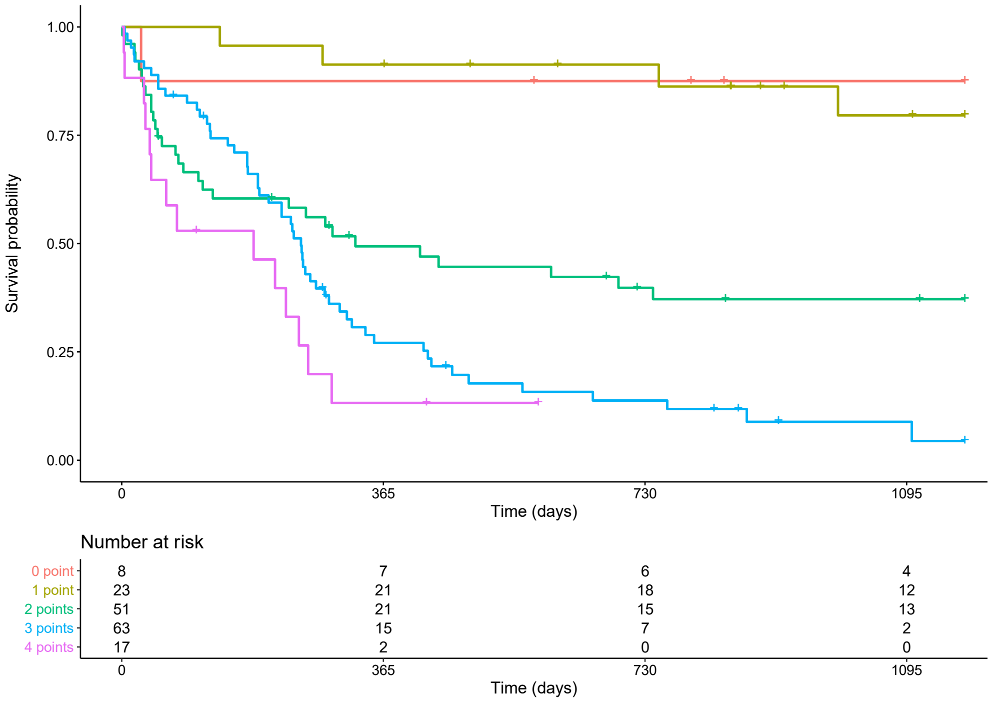

Figure S6. Kaplan-Meier survival curve according to the points allotted to patients in Table 4 for the training cohort (n = 162). p-values were determined with log-rank test. P-values were computed between curves "0" and "1 point" ($p = 0.8$); "1" and "2 points" ($p < 0.001$); "2" and "3 points" ($p = 0.01$); and "3" and "4 points" ($p = 0.09$). Therefore, patients with 0 or 1 point were allocated to Group A; patients with 2 points were allocated to Group B; and patients with 3 or 4 points were allocated to Group C.
